# A Conditional Knockout Toolkit for *Caenorhabditis elegans* Based on the *Cre/loxP* Recombination

**DOI:** 10.1371/journal.pone.0114680

**Published:** 2014-12-04

**Authors:** Eriko Kage-Nakadai, Rieko Imae, Yuji Suehiro, Sawako Yoshina, Sayaka Hori, Shohei Mitani

**Affiliations:** 1 Department of Physiology, Tokyo Women’s Medical University School of Medicine, Tokyo, Japan; 2 The OCU Advanced Research Institute for Natural Science and Technology, Osaka City University, Osaka, Japan; Brown University/Harvard, United States of America

## Abstract

Conditional knockout (cKO) based on site-specific recombination (SSR) technology is a powerful approach for estimating gene functions in a spatially and temporally specific manner in many model animals. In *Caenorhabditis elegans (C. elegans)*, spatial- and temporal-specific gene functions have been largely determined by mosaic analyses, rescue experiments and feeding RNAi methods. To develop a systematic and stable cKO system in *C. elegans*, we generated Cre recombinase expression vectors that are driven by various tissue-specific or heat-shock promoters. Validation using Cre-mediated fluorescence protein inactivation or activation systems demonstrated successful Cre-dependent *loxP* excision. We established a collection of multi-copy Cre transgenic strains for each evaluated vector. To evaluate our Cre/*loxP*-based cKO system, we generated *sid-1* deletion mutants harboring floxed *sid-1* single-copy integration (SCI) using ultraviolet trimethylpsoralen (UV/TMP) methods. *sid-1* mutants that were rescued by the floxed *sid-1* SCI were then crossed with the *Pdpy-7::Cre* strain for cKO in the hypodermis. The *sid-1* cKO animals were resistant to *bli-3* RNAi, which causes the Bli-phenotyple in the hypodermis, but they were sensitive to *unc-22* RNAi, which leads to twitching of the body wall muscle. Our system, which is based on the combination of a transgenic Cre collection, pre-existing deletion mutants, and UV/TMP SCI methods, provided a systematic approach for cKO in *C. elegans*.

## Introduction

The spatial and temporal control of gene inactivation is an essential approach for assessing gene functions in multicellular organisms. Conditional knockout (cKO) strategies based on site-specific recombination (SSR) technologies have been intensively used thus far. A cKO resource that covers 9,000 targeted alleles has been produced in mouse embryonic stem cells [1], and it has promoted systematic studies of mouse genes. In *Caenorhabditis elegans (C. elegans)*, various genetic approaches such as mosaic analyses using extrachromosomal arrays [2], transgenic rescue driven by cell-type specific or heat-shock promoters, and feeding RNAi methods [3], have been largely used to investigate spatial- and temporal-specific gene functions. Recently, conditional genome editing methods using the TALENs or CRISPR-cas9 systems have been developed [4]–[6]. The somatic TALEN or CRISPR-Cas9 provides a quick approach to generate cKOs. These somatic genome editing methods are, however, heterogeneous and not heritable.

*C. elegans* Deletion Mutant Consortium and the worm community established a collection of deletion or null mutation in approximately 7,000 genes [7]. The large mutant collection could be a powerful toolkit for systematic cKO when combined with single-copy integration (SCI) of floxed transgenes. Frøkjaer-Jensen *et al*. have developed an elegant SCI technique based on targeting integrations using Mos1 transposons [8]. We previously developed an alternative single/low-copy integration, ultraviolet trimethylpsoralen (UV/TMP) method [9], [10] that was based on the random integration of transgenes into the chromosomes from multi-copy extrachromosomal arrays, which should be familiar to most *C. elegans* researchers. Although our method exhibited lower frequency of SCI when compared to MosSCI, we adopted *vps-45*/*ben-1* as a positive/negative selection marker that provides sharp and efficient selections [9].

Site-specific recombination systems, such as the Cre/*loxP* [11] and FLP/*FRT* [12] systems, have been used to control gene expression/inactivation [13]. The Cre/*loxP* system that has an optimum temperature of 37°C and greater [14] has been widely used for genetic manipulation in mammals. The optimum temperature for FLP recombinase is 30°C [14], which is closer to growth temperature of invertebrates. *C. elegans* has a growth temperature of around 20°C, and FLP/*FRT*-mediated gene expression systems have been developed [15]–[17]. Meanwhile, several studies have demonstrated that Cre-mediated *loxP* excision successfully occurs [9], [18], [19].

In this study, we established a collection of Cre-expression vectors and transgenic strains that are driven by tissue-specific and heat-shock promoters. We also generated tissue-specific knockout mutants in a combination of Cre-transgenic lines, deletion mutants, and UV/TMP methods, and demonstrated the cKO of the targeted gene.

## Materials and Methods

### Strains

*C. elegans* strains were cultured using standard techniques [20]. The wild-type strain Bristol N2 was obtained from the Caenorhabditis Genetics Center. The *sid-1* deletion mutant strain was obtained from the UV/TMP mutagenized library, which has been previously described [21], and this strain was identified by PCR amplification with primers spanning the deletion region of *sid-1(tm2700)V*. The mutants were backcrossed five times with N2, and the *ben-1(tm234)III*;*sid-1(tm2700)V*;*vps-45(tm246)X* mutants were generated by crossing *sid-1(tm2700)* into *ben-1(tm234)*;*vps-45(tm246)* [9]. The primers that were used for PCR genotyping were as follows: tm2700_1^st^round, 5′-CAGTGGCTTCACCTGTCTTA-3′, 5′-CGTACATTCGCCGGCACAGT-3′; tm2700_2^nd^round, 5′-CGACGTTAAACACATCTCAC-3′, 5′-CGCCGGCACAGTTATCAGAT-3′; tm234_1^st^round, 5′-ACGTGGGAATGGAACCATGT-3′, 5′-TCTCCATTTCCTCTTCCTCC-3′; tm234_2^nd^round, 5′-CTCCGGACATTGTAACGGAA-3′, 5′-CCCTCCATTTGAAAGAGTCC-3′; and tm246, 5′-CGCAATTGGATACTACTTGT-3′, 5′-TCTCCTGCTCTACTTCTGCT-3′.

### Constructs and multi-copy transgenic strains

*pFX_HBG_Lw_dpy-30p_NLS_GFP*, a floxed pan-NLS::GFP plasmid, was constructed as previously described [10]. Briefly, approximately 1.2 kbp of the upstream genomic region of the *dpy-30* gene was subcloned into *pPD96.04*, and the *Pdpy-30::NLS::GFP::LacZ* sequence was amplified from the plasmid and then subcloned into *pFX_HBG_Lw* [9]. *pFX_HBG_ Lw_sid-1* was constructed by subcloning the *sid-1* genomic fragment into the BamHI/NotI sites of *pFX_HBG_Lw.* The positive selection marker (*Peft-3::vps-45*) and negative selection marker plasmid (*pGEMT_ben-1(+)*) were constructed as described in our previous study [9]. The positive/negative selection marker plasmids can be provided upon request. To generate *tmEx3452* transgenic animals, 60 ng/µl each of *Peft-3::vps-45*, *pGEMT_ben-1*(+) and *pFX_HBG_Lw_sid-1* and 20 ng/µl of the injection marker *P_myo-2_::venus* were co-injected into *ben-1(tm234);sid-1(tm2700);vps-45(tm246)*. Cre recombinase cDNA that was amplified from AxCANCre (TaKaRa) or NLS::Cre cDNA that was amplified from pPGK-Cre-bpA (Addgene) were cloned into the NotI/BglII sites of the pFX vector [22]. The genomic fragments for the tissue-specific and heat-shock promoters that are listed in Table S1 were amplified from the N2 genome and cloned 5′ into the Cre or NLS::Cre in the pFX_Cre or pFX_NLS::Cre. The Cre multi-copy integration strains were generated as previously described [23], and the injection markers are listed in Table S1. Each strain was outcrossed twice with N2.

### Single/low-copy integration by UV/TMP methods

The UV/TMP methods were conducted as previously described [10] with some modifications. Mixtures of young adults and L4 larvae of *ben-1(tm234); sid-1(tm2700); vps-45(tm246); tmEx3452[floxed sid-1]* animals were treated at 0.5 µg/ml TMP in M9 buffer and then irradiated with 365 nm UV at 200 J/m^2^ at room temperature. UV/TMP-treated animals were plated onto nematode growth media (NGM) agar dishes that were seeded with *Escherichia coli* OP50 and allowed to lay eggs at 20°C. After 24 h, the P0 adults and newly hatched F1 larvae, which were possibly derived from the eggs that were fertilized before the UV/TMP treatment, were washed off using M9 buffer. The eggs remaining on the plates were incubated at 20°C for another 24 h, until the F1 animals hatched. The hatching rate was approximately 80%. The F1 animals were cultured at 20°C for 2 days; after which the majority of *ts*-rescued F1 animals developed into L4 larvae (positive selection). The animals were harvested, washed three times with M9 buffer and plated onto OP50-seeded modified NGM plates (containing 8-fold Bacto Peptone and 10 µg/ml of benzimidazole (Wako)) (positive/negative selection). The worms were then cultured at 20°C on benzimidazole-containing NGM plates for 1–2 weeks. The transformed animals were cloned and further selected by PCR, which amplified intact floxed *sid-1* transgene.

### Quantitative PCR

The genomic DNA was isolated from adult animals using the DNeasy Tissue and Blood kit (QIAGEN). A quantitative PCR (qPCR) analysis was performed in a 7500 Real-time Thermal cycler (Applied Biosystems) using the Power SYBR master mix (Applied Biosystems) with the following parameters: 95°C for 10 min and 40 cycles of 95°C for 5 s, 58°C for 10 s and 72°C for 34 s. The primers for *ama-1*, *vps-45*, *ben-1, sid-1* were designed within an exon for each gene using the Primer3 software. The utilized primers were as follows: qPCR#ama-1_forward, AGATGGACCTCACCGACAAC; qPCR#ama-1_reverse, 5′-CTGCAGATTACACGGAAGCA-3′; qPCR#vps-45_forward, 5′-TGCGTGAGGTTCAAGAAGTG-3′; qPCR#vps-45_reverse, 5′-AACAGCTGGAGCCTTTTTCA-3′; qPCR#ben-1_forward, 5′-TTGGCTCCGATTTGATTACC-3′, qPCR#ben-1_reverse, 5′-TGTTTCCCCTCTACGTGACC-3′, qPCR#sid-1_forward, 5′- ACTGACGGAAAACTGCTCAATC-3′; and qPCR#sid-1_reverse, 5′- AAAGCCTACCGCCTATCCTG-3′.

The data were normalized to the *ama-1* gene, and the copy number of each transgene was calculated by comparing it to the amount of wild-type N2 endogenous genes.

### Bacterial feeding RNAi

Feeding RNAi was conducted as previously described [3] using RNAi clones from the Ahringer Library. L3 animals were transferred to RNAi plates and cultured at 20°C. RNAi phenotypes were examined 48 h later. For *bli-3* RNAi, the Bli phenotype was examined and for *unc-22* RNAi, the twitch of body wall muscle was analyzed.

### Microscopy

Differential interference contrast and fluorescence images were obtained using a BX51 microscope that was equipped with a DP30BW CCD camera (Olympus Optical Co., Ltd).

## Results and Discussion

### Evaluation of Cre expression vectors using floxed pan-NLS::GFP SCI

**Figure 1:**
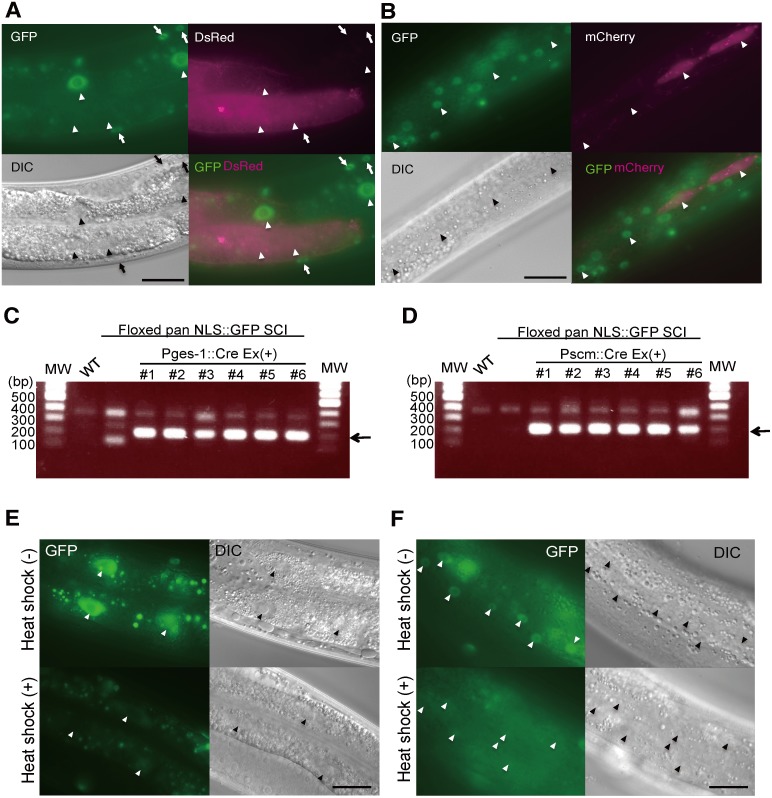
Cre-mediated floxed GFP inactivation. (A) *Pges-1::Cre* was injected into *tmIs942[floxed Pdpy-30::NLS::GFP::LacZ SCI]* along with the *Pges-1::DsRed* marker. The arrowheads indicate the nuclei of the intestinal cells. The nucleic GFP signals were eliminated in the DsRed-labeled intestinal cells. The arrows indicate the nuclei of the body wall muscle in which the GFP signals were observed. (B) *Pscm::Cre* was injected into *tmIs942[floxed Pdpy-30::NLS::GFP::LacZ SCI]* along with the *Pscm::mCherry* marker. The arrowheads indicate the nuclei of the seam cells. The nucleic GFP signals were selectively eliminated in the seam cells expressing mCherry. Scale bars = 20 µm. (C) DNA excision was examined by PCR using primers spanning the floxed region. The PCR produced 200 bp-band when the floxed region was excised. Bands of 200 bp were specifically observed in Ex animals harboring *Pges-1::Cre* (C) and *Pscm::Cre* (D), but not in the wild-type N2 or *tmIs942* parental strain. Six Ex(+) animals were analyzed for each strain. (E, F) The NLS::GFP signals were diminished in the intestine (E) and the hypodermis (F) depending on the heat-shock treatment. The arrowheads indicate the nuclei of the intestinal cells (E) and hypodermal cells (F). Scale bars = 20 µm.

Although several studies have demonstrated Cre/*loxP*-mediated gene excision in *C. elegans*, a systematic characterization has not been reported. We previously generated strains bearing the SCI of floxed *Pdpy-30::nuclear localization signal::green fluorescent protein (NLS::GFP)* transgenes (referred to as “floxed pan-NLS::GFP SCI”) [10]. To evaluate the tissue-specific Cre/*loxP*-mediated gene excision, a Cre expression vector that was driven by the *ges-1* promoter was constructed, and injected into the floxed pan-NLS::GFP SCI with *Pges-1::DsRed.* The Ex animals that were obtained were cultured at 25°C until the L4 or young adult stage and then analyzed. We observed that NLS::GFP had disappeared in the intestinal cells that were expressing DsRed but remained in the intestinal cells not expressing the DsRed marker (Fig. 1A, referred to as “GFP-OFF” in Table 1). The nucleic GFP signals were not affected in other tissues such as the body wall muscle (Fig. 1A). Similarly, when *Pscm::Cre* was expressed, the GFP signals were selectively diminished in the nuclei of the seam cells that expressed marker fluorescence proteins (Fig. 1B, Table 1). DNA excision was examined by PCR, in which a 200-bp band was produced only when the floxed region was excised. Products of the 200 bp were observed selectively in *Pges-1::Cre* and *Pscm::Cre* transgenic animals (Fig. 1C, D), and the results indicated that tissue-specific Cre/*loxP*-mediated gene inactivation occurred. Because Cre recombinase does not seem to contain NLS, artificial NLS-added Cre recombinase was used in the previous report [24]. We constructed *Pges-1::NLS::Cre* and determined its activity, and the results showed that *Pges-1::NLS::Cre* exhibited almost the same activity as *Pges-1::Cre* (data not shown). We further assessed the Cre/*loxP*-mediated gene inactivation at lower temperatures such as 20°C and 15°C. In many cases, Cre/*loxP*-mediated GFP elimination occurred at the same level that was found at 25°C. In contrast, *Pmyo-3::Cre* caused GFP elimination at 15°C but not at 25°C (data not shown), although the optimum temperature of Cre recombinase was 37°C and greater [14]. Because the growth rate of *C. elegans* is slower at lower temperatures, such conditions might offer enough time to react to Cre/*loxP* recombination and GFP turnover.

**Table 1:** Cre recombinase expression vectors driven by tissue specific and heat-shock promoters and transgenic strains.

Promoter	Expression	Excision(PCR)	Excision(GFP-OFF)	Excision(Venus-ON)	Cre multi-copyIntegration strains
*ges-1*	intestine	Yes	Yes	Yes	*tmIs1000-1002*
*myo-2*	pharynx	Yes	No	Yes	*tmIs1076-1078*
*myo-3*	BWM	Yes	Yes/No	Yes	*tmIs1058,1087,1097*
*scm*	seam cell	Yes	Yes	NA	*tmIs969-972*
*dpy-7*	hypodermis	Yes	Yes	NA	*tmIs1027-1029*
*unc-122*	coelomocyte	Yes	No	NA	*tmIs0960-0963*
*hsp-16.2*	heat shock	Yes	Yes	NA	*tmIs1054,1055,1057*
*rgef-1*	pan-neuron	Yes	No	Yes	*tmIs777-778*
*snb-1*	pan-neuron	NA	NA	Yes	-
*unc-4*	DA, VA	NA	No	Yes	*tmIs1068-1070*
*unc-25*	DD	NA	No	Yes	*tmIs1072,1073*
*eat-4*	glutamatergicneurons	NA	No	Yes	*tmIs1062,1064,1065*
*che-2*	sensoryneurons	NA	NA	Yes	*tmIs1092,1094-1096*
*ttx-3*	AIY	NA	NA	Yes	*tmIs1091*
*mec-4*	touchreceptorneurons	NA	NA	Yes	*tmIs1074,1075*
*fig-1*	glia	Yes	No	Yes	*tmIs1024-1026*
*lag-2*	DTC	Yes	Yes	NA	*-*

NA = not analyzed.

Next, we examined whether Cre/*loxP*-mediated gene inactivation can be temporally controlled. A Cre expression vector driven by the *hsp-16.2* promoter was constructed and injected into the floxed pan-NLS::GFP SCI. The Ex animals were heat-shocked at 33°C for 1 h and cultured at 15°C until the adult stage. As a result, the NLS::GFP signals were diminished depending on the heat-shock (Fig. 1E, F). Taken together, our data demonstrate that Cre/*loxP*-mediated gene inactivation is spatially and temporally controlled in *C. elegans*.

### Establishment and validation of a collection of Cre expression vectors and transgenic strains

**Figure 2:**
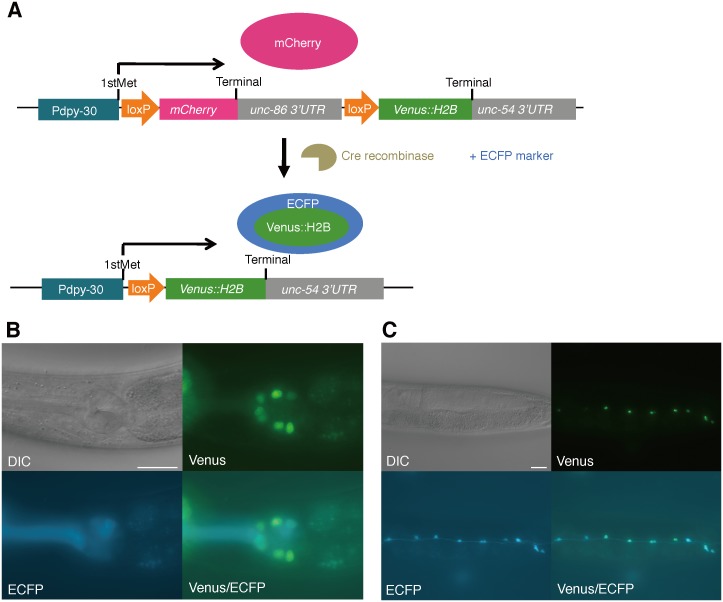
Cre-mediated Venus::H2B expression. (A) Schematic illustration of the *Pdpy-30<mCherry<venus::H2B* tester vector. Cell type-specific Cre expression vectors were co-injected with the tester vector and corresponding cell type ECFP markers. Venus::H2B is co-expressed with cytosolic ECFP in the cells where Cre/*loxP* recombination occurs. (B) Venus::H2B expression was detected with ECFP in pharyngeal cells of the *Ex[Pdpy-30<mCherry<venus::H2B;Pmyo-2::Cre;Pmyo-2::ECFP]* animals (B) and in ventral nerve cord neurons of the *Ex[Pdpy-30<mCherry<venus::H2B;Punc-4::Cre;Punc-4::ECFP]* animals (C). Scale bars = 20 µm.

For systematic experiments, we constructed a collection of Cre expression vectors that were driven by the promoters that are listed in Table 1. The vectors were validated by PCR and GFP elimination in the floxed pan-NLS::GFP SCI. Unexpectedly, GFP elimination was not observed in many cases, although Cre/*loxP*-mediated DNA excision was detected by PCR in all of the tested vectors (Table 1). We also noticed that NLS::GFP was abolished in some tissues, such as the intestine and hypodermis, but not in the other tissues, such as the neurons and pharynx (data not shown). We speculated that nucleic GFP might persist in these tissues even if the DNA is excised out. To visualize Cre/*loxP*-excision more clearly, we constructed the tester vector *Pdpy-30<mCherry<venus::H2B,* which expresses Venus::H2B when floxed mCherry was excised (Fig. 2A). Cre expression vectors were co-injected with the tester vector and corresponding enhanced cyan fluorescent protein (ECFP) markers, and then examined to determine whether they cause Venus::H2B expression. As a result, Venus::H2B expression was detected in the nuclei of the pharyngeal cells which express ECFP in *Ex[Pdpy-30<mCherry<venus::H2B;Pmyo-2::Cre;Pmyo-2::ECFP]* transgenic animals (Fig. 2B, referred as “Venus-ON” in Table 1). Similarly, Venus::H2B was observed in the ventral nerve cord neurons of *Punc-4::Cre* transgenic animals (Fig. 2C, Table 1). Venus::H2B was expressed by all of the tested Cre expression vectors (Table 1). In certain experiments, Venus::H2B was ectopically observed; however, it mostly exhibited specific expression patterns. For example, Venus::H2B was expressed by *Peat-4::Cre* not only in the head and pharyngeal neurons (Fig. S1A), which are consistent with reports that *eat-4* is expressed in glutamatergic neurons, but also in the hypodermal cells (Fig. S1B). Similarly, Venus::H2B was occasionally found in the intestine in *Psnb-1::Cre* animals (Fig. S1C, D). Unknown or embryonic/developmental expression driven by promoters may cause the ectopic Cre/*loxP* recombination. Another possibility is that rearrangement in the extrachromosomal arrays may lead to the unexpected reaction.

For stable Cre/*loxP* experiments, we generated a series of Cre multi-copy integration strains (Table 1, Table S1). Multiple strains for each promoter eliminated conflicts in the Cre-insertion sites and allowed genes to be analyzed. All of the listed strains and plasmids are available upon request. For *Snb-1p::Cre* and *lag-2::Cre*, only the plasmids are available.

### Floxed *sid-1* SCI using UV/TMP methods

**Figure 3:**
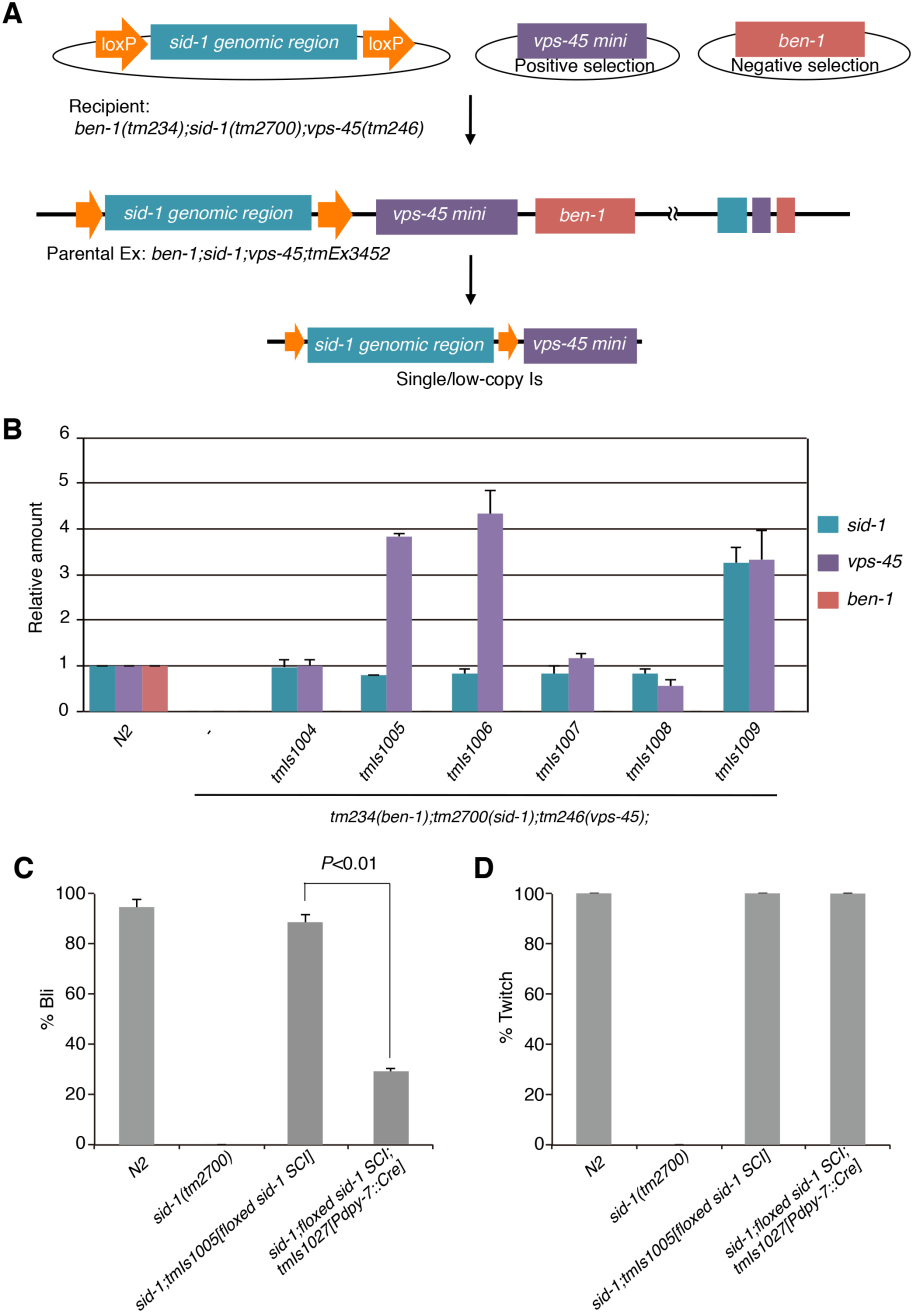
*sid-1* was conditionally knocked out by Cre expression. (A) Schematic overview of floxed *sid-1* SCI by UV/TMP methods. The floxed sid-1 plasmid was co-injected with positive/negative selection marker plasmids into the recipient strain *ben-1(tm234);sid-1(tm2700);vps-45(tm246)* to establish parental Ex strains. The obtained Ex strain, *ben-1(tm234);sid-1(tm2700);vps-45(tm246);tmEx3452* was treated with a TMP solution followed by UV irradiation. Single-low-copy integration strains were selected under the positive/negative selection condition. (B) The relative amounts of the *sid-1*, *vps-45,* and *ben-1* genes were determined using quantitative PCR, and the levels of the genes (normalized to the *ama-1* gene) are presented as ratios to the N2 control. The error bars represent the SE of three independent experiments. (C, D) Percentages of animals exhibiting the Bli phenotype in the bli-3 RNAi (C), and the twitch of body wall muscle in the unc-22 RNAi (D). The error bars represent the SE of three independent experiments. The statistical analysis was performed using *t*-test.

To validate our system, we performed cKO on the *sid-1* gene. We selected *sid-1* because the systemic RNAi-defective phenotype of the mutants is clear and can be assessed throughout the entire body except for in the neurons. First, we generated floxed *sid-1* SCI using UV/TMP (Fig. 3A). Approximately 10,000 P0 animals from the parent Ex strain (*tm234;tm2700;tm246;tmEx3452[floxed sid-1])* were treated with UV/TMP, and approximately 200,000 F1 animals were collected and cultured under the selection conditions. As a result, 47 stable transformants were obtained. The isolated transformants were further selected by PCR, which resulted in 6 integrated strains with the *floxed sid-1* transgene. The integration frequency (integrants/P0 animals) was 0.06%. A qPCR analysis of the *sid-1* sequence showed that the five strains, *tmIs1004, tmIs1005, tmIs1006, tmIs1007*, and *tmIs1008* had a single-copy integration of the floxed *sid-1* transgene (Fig. 3B). In contrast, *tmIs1009* contained a low-copy (3 copies) of the transgenes. A qPCR analysis of the *vps-45* revealed a single copy insertion of the *vps-45 *mini gene in *tmIs1004, tmIs1007* and *tmIs1008* and several-copy insertion in *tmIs1005, tmIs1006* and *tmIs1009* (Fig. 3B). When examining the *tmEx3452* parental strain, it was revealed that the parental Ex strain contained 32, 139 and 52 copies of transgenes for *sid-1*, *vps-45* and *ben-1*, respectively. The *ben-1* gene was nearly undetectable in the integrant strains, which was expected (Fig. 3B). In this experiment, we successfully obtained multiple single-copy integrations of the floxed *sid-1*, the size of which is approximately 10 kbp.

### Tissue-specific knockout for the *sid-1* gene

The obtained *ben-1(tm234);sid-1(tm2700);vps-45(tm246)* mutants harboring the floxed *sid-1* SCI were crossed with N2 to generate *sid-1*; floxed *sid-1* SCI. The RNAi of *elt-2* (intestine), *unc-22* (body wall muscle), *bli-3* (hypodermis), and *pos-1*(germline) indicated that the RNAi-resistant phenotype of the *sid-1* mutant was rescued by the floxed *sid-1* SCI (data not shown). *sid-1*; floxed *sid-1* SCI was then crossed into Cre multi-copy transgenic strains. When the *sid-1(tm2700);tmIs1005[floxed sid-1 SCI];tmIs1027[Pdpy-7::Cre]* animals were examined by *bli-3* RNAi, the percentage of animals exhibiting the Bli-phenotype was significantly reduced, demonstrating that *sid-1* was at least partially inactivated in the hypodermis (Fig. 3C). In addition, the *sid-1(tm2700);tmIs1005[floxed sid-1 SCI];tmIs1027[Pdpy-7::Cre]* animals were fully sensitive to *unc-22* RNAi (Fig. 3D). These results indicated that the tissue-specific inactivation of *sid-1* occurred in our system.

## Conclusion

In this study, we provide an approach for *C. elegans* cKO based on the combination of a Cre-transgenic collection, pre-existing deletion mutants, and UV/TMP SCI methods. We established a series of Cre expression vectors and transgenic strains driven by tissue-specific and heat-shock promoters and assessed their activities using Cre/*loxP*-mediated gene activation and inactivation systems. We also showed that Cre/*loxP* recombination occurred at 15°C, 20°C, and 25°C, which were within the culture temperature range of *C. elegans*. It is recommended to confirm that the targeted proteins are certainly abolished in Cre/*loxP* experiments, because we experienced the cases in which the targeted protein persisted despite the excision of the targeted floxed sequence. We further obtained *sid-1* deletion strains harboring the single-copy integration of the floxed *sid-1* transgene using UV/TMP methods and showed that the floxed *sid-1* was conditionally inactivated by Cre expression, demonstrating that UV/TMP methods can be used to generate single-copy integration for cKO.

Researchers have now several options, the somatic genome editing using the CRISPR-Cas9 or TALENs, and SSR using the Cre/*loxP* or FLP/*FRT*, when generating cKOs. Of these, the somatic CRISPR-Cas9 seems to be the most rapid strategy. This technology enables the analysis of cKO phenotypes within 1 week from the microinjection of Cas9 and sgRNA plasmids [6]. Although the CRISPR-Cas9 system yields higher mutation efficiencies than TALENs in many cases, TALENs may be an alternative strategy to edit a locus that is challenging for the somatic CRISPR-Cas9 method [4], [6]. However, SSR using the Cre/*loxP* or FLP/*FRT* should be used in preference when consistent genotypes and phenotypes are desirable, because cKOs using the somatic genome editing methods are heterogeneous and not heritable. The Cre/*loxP* or FLP/*FRT* methods are technically both applicable to *C. elegans* cKO, although FLP/*FRT* -mediated gene inactivation in *C. elegans* has not been reported, to our knowledge. We demonstrated a systematic gene inactivation using the *Cre/loxP* in this study. A toolkit for the *Cre/loxP* system will promote efficient and convenient cKO strategies for *C. elegans* researchers.

## Supporting Information

Figure S1 Ectopic Venus::H2B expression occasionally observed. (A, B) Venus::H2B expression was detected with ECFP in the head and pharyngeal neurons of the *Ex[Pdpy-30<mCherry<venus::H2B;Peat-4::Cre;Peat-4::ECFP]* animals (A), but occasionally detected in the hypodermal cells (B). (C, D) The Venus::H2B was detected in pan-neurons of the *Ex[Pdpy-30<mCherry<venus::H2B;Psnb-1::Cre;Psnb-1::ECFP]* animals (C) but occasionally expressed in the intestinal cells (D). The arrowheads indicate ectopic expressions.
(TIFF)

Table S1 Detailed information on Cre expression vectors and transgenic strains.
(XLSX)

## References

[1] Skarnes WC, Rosen B, West AP, Koutsourakis M, Bushell W, et al (2011) A conditional knockout resource for the genome-wide study of mouse gene function. Nature 474:337–342. 21677750 10.1038/nature10163 PMC3572410

[2] Mello C, Fire A (1995) DNA Transformation. 8531738

[3] Kamath RS, Martinez-Campos M, Zipperlen P, Fraser AG, Ahringer J (2001) Effectiveness of specific RNA-mediated interference through ingested double-stranded RNA in Caenorhabditis elegans. Genome Biol 2:RESEARCH0002. 11178279 10.1186/gb-2000-2-1-research0002 PMC17598

[4] Cheng Z, Yi P, Wang X, Chai Y, Feng G, et al (2013) Conditional targeted genome editing using somatically expressed TALENs in C. elegans. Nat Biotechnol 31:934–937. 23955274 10.1038/nbt.2674

[5] Liu P, Long L, Xiong K, Yu B, Chang N, et al (2014) Heritable/conditional genome editing in C. elegans using a CRISPR-Cas9 feeding system. Cell Res 24:886–889. 24874953 10.1038/cr.2014.73 PMC4085767

[6] Shen Z, Zhang X, Chai Y, Zhu Z, Yi P, et al. (2014) Conditional Knockouts Generated by Engineered CRISPR-Cas9 Endonuclease Reveal the Roles of Coronin in C. elegans Neural Development. Dev Cell. 10.1016/j.devcel.2014.07.017 25155554

[7] Consortium CeDM (2012) large-scale screening for targeted knockouts in the Caenorhabditis elegans genome. G3 (Bethesda) 2:1415–1425. 23173093 10.1534/g3.112.003830 PMC3484672

[8] Frøkjaer-Jensen C, Davis MW, Hopkins CE, Newman BJ, Thummel JM, et al (2008) Single-copy insertion of transgenes in Caenorhabditis elegans. Nat Genet 40:1375–1383. 18953339 10.1038/ng.248 PMC2749959

[9] Kage-Nakadai E, Kobuna H, Funatsu O, Otori M, Gengyo-Ando K, et al (2012) Single/low-copy integration of transgenes in Caenorhabditis elegans using an ultraviolet trimethylpsoralen method. BMC Biotechnol 12:1. 22217006 10.1186/1472-6750-12-1 PMC3262153

[10] Kage-Nakadai E, Imae R, Yoshina S, Mitani S (2014) Methods for single/low-copy integration by ultraviolet and trimethylpsoralen treatment in Caenorhabditis elegans. Methods 68:397–402. 24613935 10.1016/j.ymeth.2014.02.036

[11] Austin S, Ziese M, Sternberg N (1981) A novel role for site-specific recombination in maintenance of bacterial replicons. Cell 25:729–736. 7026049 10.1016/0092-8674(81)90180-x

[12] Broach JR, Guarascio VR, Jayaram M (1982) Recombination within the yeast plasmid 2mu circle is site-specific. Cell 29:227–234. 6286142 10.1016/0092-8674(82)90107-6

[13] Gu H, Marth JD, Orban PC, Mossmann H, Rajewsky K (1994) Deletion of a DNA polymerase beta gene segment in T cells using cell type-specific gene targeting. Science 265:103–106. 8016642 10.1126/science.8016642

[14] Buchholz F, Ringrose L, Angrand PO, Rossi F, Stewart AF (1996) Different thermostabilities of FLP and Cre recombinases: implications for applied site-specific recombination. Nucleic Acids Res 24:4256–4262. 8932381 10.1093/nar/24.21.4256 PMC146240

[15] Davis MW, Morton JJ, Carroll D, Jorgensen EM (2008) Gene activation using FLP recombinase in C. elegans. PLoS Genet 4:e1000028. 18369447 10.1371/journal.pgen.1000028 PMC2265415

[16] Voutev R, Hubbard EJ (2008) A “FLP-Out” system for controlled gene expression in Caenorhabditis elegans. Genetics 180:103–119. 18723890 10.1534/genetics.108.090274 PMC2535667

[17] Hubbard EJ (2014) FLP/FRT and Cre/lox recombination technology in C. elegans. Methods 68:417–424. 24874786 10.1016/j.ymeth.2014.05.007 PMC4210360

[18] Hoier EF, Mohler WA, Kim SK, Hajnal A (2000) The Caenorhabditis elegans APC-related gene apr-1 is required for epithelial cell migration and Hox gene expression. Genes Dev 14:874–886. 10766743 PMC316495

[19] Flavell SW, Pokala N, Macosko EZ, Albrecht DR, Larsch J, et al (2013) Serotonin and the neuropeptide PDF initiate and extend opposing behavioral states in C. elegans. Cell 154:1023–1035. 23972393 10.1016/j.cell.2013.08.001 PMC3942133

[20] Brenner S (1974) The genetics of Caenorhabditis elegans. Genetics 77:71–94. 4366476 10.1093/genetics/77.1.71 PMC1213120

[21] Gengyo-Ando K, Mitani S (2000) Characterization of mutations induced by ethyl methanesulfonate, UV, and trimethylpsoralen in the nematode Caenorhabditis elegans. Biochem Biophys Res Commun 269:64–69. 10694478 10.1006/bbrc.2000.2260

[22] Gengyo-Ando K, Yoshina S, Inoue H, Mitani S (2006) An efficient transgenic system by TA cloning vectors and RNAi for C. elegans. Biochem Biophys Res Commun 349:1345–1350. 16979594 10.1016/j.bbrc.2006.08.183

[23] Mitani S (1995) Gnetic regulation of *mec-3* gene expression implicated in the specification of the mechanosensory neuron cell types in *Caenorhabditis elegans* . Develop Growth Differ 37:551–557. 10.1046/j.1440-169X.1995.t01-4-00010.x 37281420

[24] Macosko EZ, Pokala N, Feinberg EH, Chalasani SH, Butcher RA, et al (2009) A hub-and-spoke circuit drives pheromone attraction and social behaviour in C. elegans. Nature 458:1171–1175. 19349961 10.1038/nature07886 PMC2760495

