# Supplementary figures and images for: A Conditional Knockout Toolkit for Caenorhabditis elegans Based on the Cre/loxP Recombination
Source: PLoS One. 2014 Dec 4;9(12):e114680. doi: 10.1371/journal.pone.0114680 (PMC4256423; doi:10.1371/journal.pone.0114680)

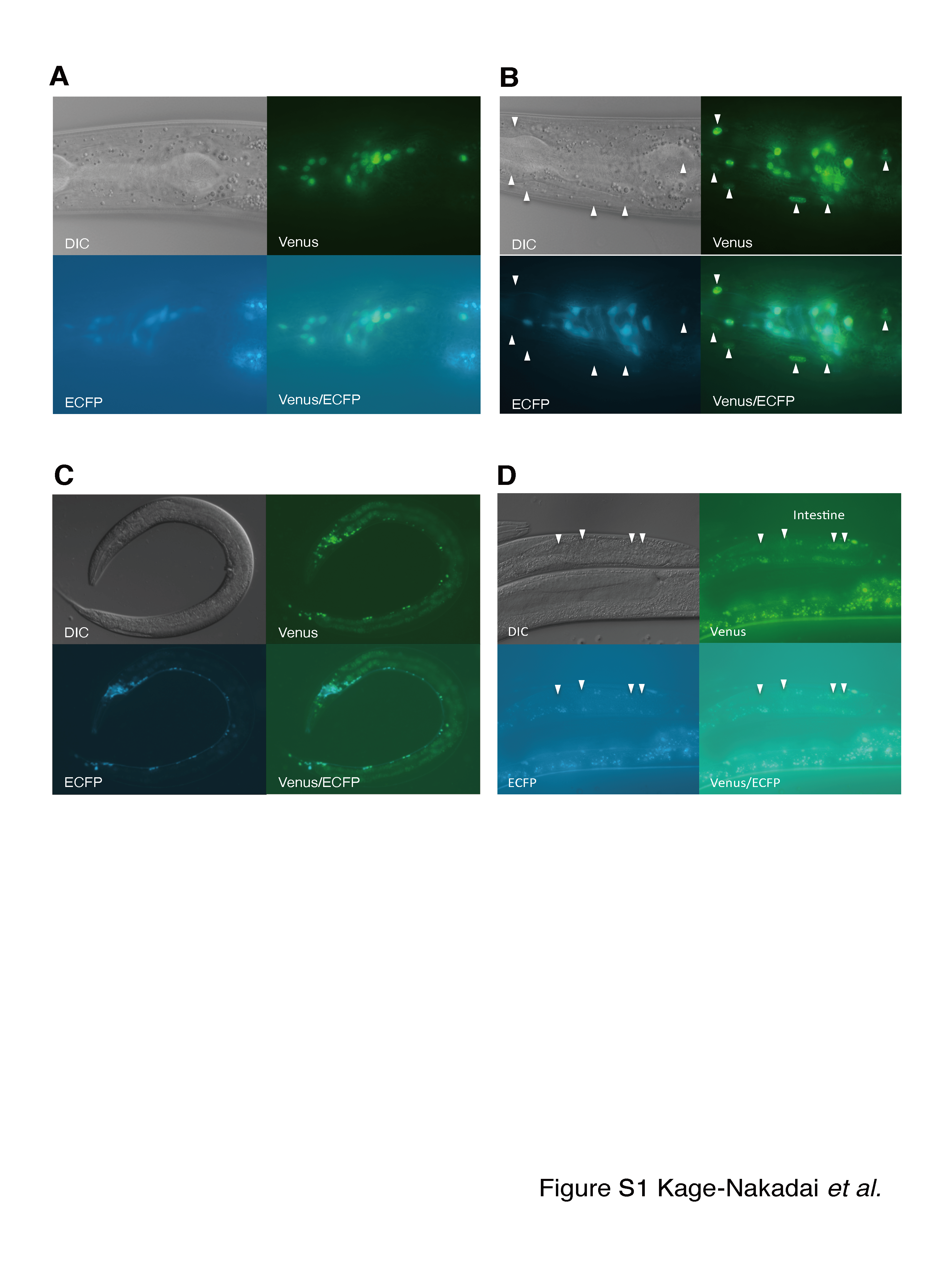

Supplement: Figure S1 — Ectopic Venus::H2B expression occasionally observed. (A, B) Venus::H2B expression was detected with ECFP in the head and pharyngeal neurons of the Ex[Pdpy-30<mCherry<venus::H2B;Peat-4::Cre;Peat-4::ECFP] animals (A), but occasionally detected in the hypodermal cells (B). (C, D) The Venus::H2B was detected in pan-neurons of the Ex[Pdpy-30<mCherry<venus::H2B;Psnb-1::Cre;Psnb-1::ECFP] animals (C) but occasionally expressed in the intestinal cells (D). The arrowheads indicate ectopic expressions. (TIFF) [file pone.0114680.s001.tiff]
